# Novel Genes Participating in the Formation of Prismatic and Nacreous Layers in the Pearl Oyster as Revealed by Their Tissue Distribution and RNA Interference Knockdown

**DOI:** 10.1371/journal.pone.0084706

**Published:** 2014-01-15

**Authors:** Daisuke Funabara, Fumito Ohmori, Shigeharu Kinoshita, Hiroki Koyama, Saeri Mizutani, Ayaka Ota, Yuki Osakabe, Kiyohito Nagai, Kaoru Maeyama, Kikuhiko Okamoto, Satoshi Kanoh, Shuichi Asakawa, Shugo Watabe

**Affiliations:** 1 Graduate School of Bioresources, Mie University, Tsu, Mie, Japan; 2 Graduate School of Agricultural and Life Sciences, The University of Tokyo, Bunkyo, Tokyo, Japan; 3 Pearl Research Laboratory, K. Mikimoto & Co., Ltd., Shima, Mie, Japan; 4 Mikimoto Pharmaceutical Co., Ltd., Ise, Mie, Japan; 5 Kitasato University School of Marine Bioscience, Sagamihara, Kanagawa, Japan; Georg August University of Göttingen, Germany

## Abstract

In our previous publication, we identified novel gene candidates involved in shell formation by EST analyses of the nacreous and prismatic layer-forming tissues in the pearl oyster *Pinctada fucata*. In the present study, 14 of those genes, including two known genes, were selected and further examined for their involvement in shell formation using the RNA interference. Molecular characterization based on the deduced amino acid sequences showed that seven of the novel genes encode secretory proteins. The tissue distribution of the transcripts of the genes, as analyzed by RT-PCR and *in situ* hybridization, was mostly consistent with those obtained by the EST analysis reported previously. Shells in the pearl oysters injected with dsRNAs targeting genes 000027, 000058, 000081, 000096, 000113 (nacrein), 000118, 000133 and 000411 (MSI60), which showed expression specific to the nacreous layer forming tissues, showed abnormal surface appearance in this layer. Individuals injected with dsRNAs targeting genes 000027, 000113 and 000133 also exhibited abnormal prismatic layers. Individuals injected with dsRNAs targeting genes 000031, 000066, 000098, 000145, 000194 and 000200, which showed expression specific to prismatic layer forming tissues, displayed an abnormal surface appearance in both the nacreous and prismatic layers. Taken together, the results suggest that the genes involved in prismatic layer formation might also be involved in the formation of the nacreous layers.

## Introduction

The shells of pearl oysters comprise two layers, termed the prismatic and nacreous layers, formed by secreting organic materials from epithelial cells in the mantle [1]. The prismatic layer is thought to be formed by the secretion of materials from the edge region of the mantle (the mantle edge) and the nacreous layer is formed from the inner part of the mantle (the pallium). The main components of shells are calcium carbonate and organic substances, such as chitin and various proteins. Crystal structures are different between the two layers: calcite is present in the prismatic layer and aragonite is found in the nacreous layer.

The accumulation of calcium carbonate as calcite and aragonite crystals was thought to be regulated by proteins secreted from the mantle. Nacrein, the first protein to be isolated from the nacreous layer, exists in both the nacreous and prismatic layers and, thus, is thought to be involved in the formation of the whole shell [2]–[4]. MSI60, a matrix protein in the nacreous layer, has several characteristic domains that are thought to be involved in the formation of this layer [5]. Pif, an acidic protein isolated from the nacreous layer, has been reported to regulate the formation of this layer [6]. Although other proteins have been isolated as matrix proteins from the prismatic and nacreous layers, it is not clear how these two layers are formed in molluscan shells [7].

In pearl culture, nuclei are inserted into the gonad gland in mother oysters together with graft tissues of the outer epithelial cells in the mantle from other individuals. The graft tissues then spread over the nuclei to develop a pearl sac that produces the nacreous layer on the nuclei [1]. The pearl sac comprises specialized tissues that form the nacreous layer and produce pearls. To elucidate the molecular mechanisms involved in the formation of the prismatic and nacreous layers, we analyzed expressed sequence tag (EST) sets from the mantle edge, pallium and pearl sac of the pearl oyster *Pinctada fucata* [8]. As a result, we identified more than 70 novel candidate genes that might be involved in the formation of the two layers.

RNA interference (RNAi) suppresses the expression of specific genes. This technique has been widely used to investigate functions of uncharacterized genes [9] and has been effective in bivalves [6], [10], [11]. For example, the injection of double stranded RNA (dsRNA) of *Pif* into the adductor muscle resulted in an abnormal appearance of the nacreous layer, as revealed by scanning electron microscopy (SEM) [6]. Furthermore, RNAi was useful for screening other candidate genes possibly involved in the formation of the nacreous layer [11].

The objective of the present study was to examine whether or not the candidate genes from the EST analysis would function in the formation of the nacreous layer. First, we determined the full-length cDNA sequences of the genes and then examined their functions in during shell formation by expression and RNAi knockdown analyses.

## Materials and Methods

### Novel Gene Candidates for Nacre Formation

We screened gene candidates involved in the formation of nacreous and prismatic layers from the EST database of the pearl oyster *P. fucata* [8] as follows. *De novo* assembly using MIRA assembler ver. 2.9.45×1 and the basic local alignment search tool (BLAST) Clust program from NCBI produced 29,682 unique gene sequences that were automatically numbered by the BLAST Clust program as 000001-029682, as described previously [8] (Table 1). Gene expression was represented as their appearance frequency, which corresponds to the total reads of a given gene against the total reads in the tissue (Table S1). Among the 29,682 gene sequences, we selected genes with over 200 reads and compared their appearance frequency among shell formation-related tissues, the pearl sac, mantle pallium and mantle edge. As a result, five genes (000081, 000098, 000113, 000118 and 000133) were found to be expressed twice as highly in the mantle pallium as in the mantle edge and pearl sac. Three (000027, 000096 and 000411) were highly expressed in the mantle pallium and pearl sac, whereas six genes (000031, 000058, 000066, 000072, 000104, 000145, 000194 and 000200) were highly expressed in the mantle edge with almost no expression in the pearl sac. BLAST searching identified genes 000113 and 000411 to be *nacrein* and *MSI60*, respectively (Table 1).

**Table 1:** Characteristics of genes analyzed in this study.

	Gene	Accession number	cDNA length (bp)	CDS	p*I* of gene product	Molecular size (kDa) of gene product	Homologus gene by BlastX				Expression patterns			Knock down phenotype^4^
							Name [Source]	Accession number	*E* value	Identity (%)	RT-PCR^1^	NGS^2^	*In situ* hybridization^3^	
Known *P. fucata* nacreous genes	Pif	AB236929.1	3387	43–3066 bp (1007 aa)	5.0	115.4	-	-	-	-	Go, M, PS	P>ME, PS	P, ME	Nacre, Prism
	Nacrein (000113)	BAA11940.1	2459	22–1365 bp (447 aa)	6.8	50.0	-	-	-	-	Go, AM, M,PS	P>ME, PS	P, ME	Nacre, Prism
	MSI60 (000411)	D86074.1	3331	50–2226 bp (738 aa)	4.9	61.7	-	-	-	-	Go, M, PS	P, PS>ME	P	Nacre
Candidate genes	000027	AB734766	604	100–504 bp (134 aa)	11.2	15.9	Putative ribosomal protein L32 [*Sipunculus nudus*]	ABW90364.1	8.00E-71	84	FM, Gi, Go, AM, M, PS	P, PS>ME	P, ME	Nacre, Prism
	000031	AB734767	776	45–584 bp (179 aa)	9.3	19.2	No hit	-	-	-	FM, Gi, Go, AM, M, PS	ME>P, PS	ME	Nacre, Prism
	000058	AB734768	1429	231–1196 bp (321 aa)	4.8	35.8	Hypothetical protein [*Xenopus tropicalis*]	XP_002934094.1	4.00E-28	29	FM, Gi, Go, AM, M	ME>P, PS	P	Nacre, Prism
	000066	AB734769	742	56–367 bp (103 aa)	8.7	12.1	No hit	-	-	-	Gi, M	ME>P, PS	ME	Nacre, Prism
	000081	AB734770	969	109–933 bp (274 aa)	9.7	28.0	Glycine-rich protein 2 [*Pinctada maxima*]	P86969.1	3.00E-44	65	M, PS	P>ME, PS	P	Nacre
	000096	BAK40911.1	561	172–498 bp (108 aa)	9.3	12.1	-	-	-	-	Go, AM, M, PS	P, PS>ME	P	Nacre
	000098	AB734771	3300 (partial)	1–3086 bp (1027 aa)	4.5	112.2	SCO-spondin like [*Xenopus tropicalis*]	XP_002942623.1	4.00E-51	30	FM, Gi, Go, AM, M, PS	P>ME, PS	ME	Nacre, Prism
	000118	AB734772	693	240–482 bp (81 aa)	8.9	9.2	Prism uncharacterized shell protein 18 [*Pinctada margaritifera*]	CCE46170.1	2.00E-22	57	Go, M, PS	P>ME, PS	P	Nacre
	000133	AB734773	805	3–701 bp (232 aa)	11.2	27.5	No hit	-	-	-	FM, Gi, Go, AM, M, PS	P>ME, PS	P, ME	Nacre, Prism
	000145	AB734774	779	72–350 bp (92 aa)	6.7	9.8	No hit	-	-	-	FM, AM, M, PS	ME>P, PS	P, ME	Nacre, Prism
	000194	AB734775	1466	68–1330 bp (420 aa)	8.4	40.8	No hit	-	-	-	M	ME>P, PS	ME	Nacre, Prism
	000200	AB734776	1370	37–516 bp (159 aa)	9.4	16.4	No hit	-	-	-	M	ME>P, PS	P, ME	Nacre, Prism

^1^ Tissue specificity determined by RT-PCR (see Figure 2).

^2^ Regional expression in mantle and pearl sac determined by next generation sequencing (see Table S1).

^3^ Regional expression in mantle determined by in situ hybridization (see Figure 3).

^4^ Shell layer affected by RNAi knockdown (see Figure 5).

Abbreviations used are: CDS, coding sequence; NGS, next generation sequencing; FM, foot muscle; Gi, gill, Go, gonad; AM, adductor muscle; M, mantle; PS, peral sac; P, pallium; ME, mantle edge.

### Molecular Characterization of Candidate Genes

We carried out molecular cloning of the candidate genes and predicted the molecular characteristics of their encoded proteins. 5′-rapid amplification of cDNA ends (RACE) was conducted to determine the full-length nucleotide sequences of the genes using total RNA purified from the mantle of live specimens of the pearl oyster harvested in the Mikimoto Pearl Farm, Mie Prefecture, Japan. Gene specific primers for RACE (Table S2) were designed based on the partial nucleotide sequences of the genes obtained from the EST analyses [8]. cDNA templates in 5′-RACE were synthesized using GeneRacer™ kit (Invitrogen, Carlsbad, CA, USA). 5′- RACE products were subcloned into the pGEM-T Easy vector (Promega, Madison, WI, USA) and sequenced with an ABI3100 Genetic Analyzer (Applied Biosystems, Foster City, CA, USA). The sequences obtained were subjected to BLAST analysis with the option of BLASTX or BLASTP, with an expected value of 1E-10, against all sequences registered in the National Center for Biotechnology Information (NCBI) database. The amino acid sequences deduced from the nucleotide sequences were subjected to analyses of their molecular characteristics, using Pfam to predict the motif structure, SignalP v4.0 to predict the presence of signal peptides and their cleavage sites, ProtParam to compute the molecular weight and theoretical p*I*, TMHMM to predict the region of transmembrane helices in proteins, NetOGlyc to predict mucin type GalNAc *O*-glycosylation sites and NetNGlyc to predict *N*-glycosylation sites [12]–[17]. All sequences determined in this study were registered in the DDBJ/EMBL/GenBank databases with the accession numbers AB734766- AB734776.

### Tissue Distribution Analysis

Adult specimens of the pearl oyster were collected from the Mikimoto Pearl Farm, Mie Prefecture, Japan. Foot muscle, gill, gonad, adductor muscle, mantle and pearl sac tissues were dissected and preserved in RNA*later* (Applied Biosystems). Total RNA was extracted from the above-mentioned tissues with an Isogen kit (Nippon Gene, Tokyo, Japan). Reverse transcription (RT)-PCR was performed with gene specific primers (Table S3) based on the full-length or partial cDNA sequences. *Pif* [6] and the elongation factor-1α gene (*EF-1α*) (DDBJ/EMBL/GenBank accession number, AB205403) were used as the positive reference and internal control, respectively. PCR was conducted as follows: denaturation at 94°C for 5 min; followed by 30 cycles at 94°C for 30 s, 55°C for 30 s and 72°C for 1 min; with a final extension step at 72°C for 7 min. PCR products were then subjected to electrophoresis through a 1% agarose gel. For *in situ* hybridization, mantle tissues containing the pallium and mantle edge were fixed in 4% (w/v) paraformaldehyde overnight, embedded in Optimal Cutting Temperature compound (Funakoshi, Tokyo, Japan), and sectioned at a thickness of 18 µm. cDNA fragments were amplified for RNA probe synthesis by RT-PCR from the mantle using gene-specific primers designed from the 3′-untranslated region (UTR) of each gene (Table S4). Sense and anti-sense digoxigenin (DIG)-labeled RNA probes were generated with T7 and SP6 RNA polymerases, respectively, from the cDNA fragments, using a DIG RNA Labeling kit (Roche Applied Science, Mannheim, Germany). Hybridization was performed at 58°C.

### RNA Interference Experiments

RNAi experiments were performed according to the method reported by Suzuki et al. (2009), with some modifications. cDNA was synthesized with a 3′-Full RACE Core Set (Takara, Otsu, Japan) using the total RNA from mantle tissues as a template. PCR was carried out to obtain the DNA fragments encoding the candidate genes using primers designed based on the sequences of these genes. *Pif* was used as a positive reference gene. The green fluorescence protein (GFP) gene was used as negative reference to verify the RNAi experiments. The T7 promoter sequence was added to the 5′ end of each primer to subject the PCR products to dsRNA synthesis with T7 RNA polymerase. dsRNAs were synthesized using a ScriptMAX™ Thermo T7 Transcription Kit (Toyobo, Osaka, Japan), according to the manual of the kit, using the cDNA clones encoding candidate and reference genes. About 40 µg of dsRNA/100 µl water of MilliQ (Merck Millipore, MA, USA) was injected into adductor muscles in live specimens of two-year-old pearl oysters. The injected individuals were reared in artificial seawater at 23°C for eight days, with feeding once a day. The surface on the inside of shells of the RNAi knockdown individuals was observed with a scanning electron microscope (SEM) S-4000 (Hitachi, Tokyo, Japan), focusing on the prismatic and nacreous layers, and the calcite-aragonite boundary region.

The effect of RNAi knockdown was validated as follows. Total RNA was extracted from the mantle of each individual eight days after injection and first-strand cDNA was synthesized as describe above. Real-time quantitative PCR (qPCR) was employed to quantify the expression levels of the target genes, *MSI60*, *nacrein*, and gene 000096. *EF-1α* was used as an internal control, as described previously [18]. qPCR was conducted using the ABI Prism 7300 Sequence Detection System (Applied Biosystems) with an SYBR premix Ex*Taq* II kit (Takara), according to the manufacturer's instructions (Table S4). The cycling parameters consisted of one cycle of 95°C for 30 s, followed by 40 cycles of 95°C for 5 s, 55°C for 30 s and 72°C for 30 s. Dissociation curves were analyzed to determine the purity of the products and the specificity of amplification. In the control, the expression level of the group injected with MilliQ water was set as 1.0. For the differential gene expression analysis among various samples, statistical analyses were performed using one-way analysis of variance (ANOVA), followed by Tukey's test in Sigma Plot 10.0 (SYSTAT, Chicago, IL, USA). Data were represented as the mean ± standard error (*n* = 3), and the differences were considered significant at *P*<0.05.

## Results

### cDNA Cloning and Molecular Characterization

**Figure 1:**
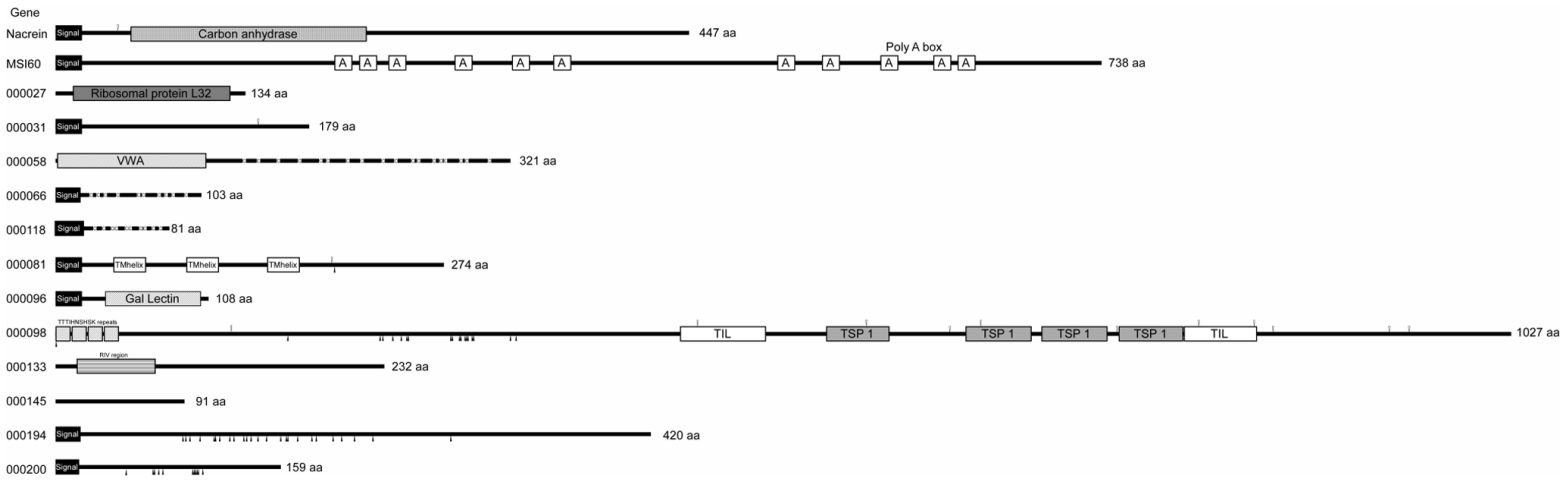
Schematic representation of motif structures and other characteristic features of proteins encoded by the novel genes analyzed in the present study. Boxes with names of motifs represent those identified by Pfam. Boxes with A in MSI60 represent the region composed of poly alanine residues. TTTIHNSHSK repeats and RIV region are newly found in the present study. The number at the right of each sequence represents that of the amino acid residue from the N terminus in the molecule. Black and white arrowheads represent the positions of putative *O*- and *N*-linked glycosylation sites predicted by NetOGlyc and NetNGlyc, respectively. Abbreviations used are: signal, signal peptide; TIL, trypsin inhibitor like cysteine rich domain; TSP 1, thrombospondin type 1 domain; TM helix, transmembrane helix; Gal Lectin, galactose binding lectin domain; VMD, von Willebrand factor type A domain. Cs in the 000204 and 000058 proteins represent cysteine residues. TTTIHNSHSK repeat in the 000098 protein means the repetitive sequence of TTIHNSHSK. The RIV region in the 000133 protein indicates the region rich in arginine, isoleucine and valine residues. Data for nacrein (accession no. BAA11940) and MSI60 (BAA20466) were cited from GenBank.

The full-length cDNA sequence of gene 000096 has been determined in our previous study [8]. The 000096 protein contained a N-terminal signal peptide and a galactose binding lectin domain (Fig. 1).

As described above, genes 000113 and 000411 were identified as known *P. fucarta* nacreous genes, *nacrein* [2] and *MSI60* [5], respectively (Table 1).

In the present study, we determined the full-length cDNA sequences of genes 000027, 000031, 000058, 000066, 000081, 000118, 000133, 000145, 000194 and 000200, and a partial cDNA sequence of gene 000098. Among them, genes 000027, 000081, 000098 and 000118 showed significant homology (*E* value of 8.00E-71∼2.00E-22) with known gene sequences, as shown in Table 1. We tried to obtain cDNA clones of genes 000072 and 000104, without success; therefore, these genes were not subjected to the subsequent RT-PCR, in situ hybridization and RNAi experiments.

It was notable that genes 000081 and 000118 showed high homology with glycine rich protein 2 and prism uncharacterized shell protein genes, respectively. The gene encoding glycine rich protein 2 was expressed in the mantle of *P. maxima* [19], whereas the prism uncharacterized shell protein was found in the shell of *P. margaritifera* [20], suggesting their direct involvement in shell formation in *Pinctada* species.

Genes 000027, 000058 and 000098 showed homology with a ribosomal protein gene of *Sipunculus nudus* [21], a hypothetical protein gene of *Xenopus tropicalis* and a SCO-spondin like gene of *X. tropicalis*, respectively (Table 1).

The remaining six genes (000031, 000066, 000133, 000145, 000194 and 000200) showed no significant homology with known database sequences. The proteins encoded by genes 000031, 000066, 000194 and 000200 had predicted N-terminal signal peptides (Fig. 1). The Pfam program predicted that the deduced amino acid sequence of gene 000058 contained a von Willebrand factor type A (VWA) domain followed by a sequence containing 18 cysteine residues (Fig. 1).

On the other hand, the translation product of gene 000194 contains a region near the C-terminus that is rich in methionine and glycine, forming the repeat sequences MGG or MGGG (Fig. 1).

The protein encoded by gene 000200 contains a region where glycine residues were continuously arranged following the signal peptide (Fig. 1).

The translation product of gene 000133 contains a characteristic region consisting of RVRRI repeats, though its function is unknown (Fig. 1).

The translation product of gene 000145 comprised 91 amino acids and a predicted molecular weight of 9.8 kDa. No characteristic structure was predicted in the molecule.

### Tissue Distribution of Transcripts

**Figure 2:**
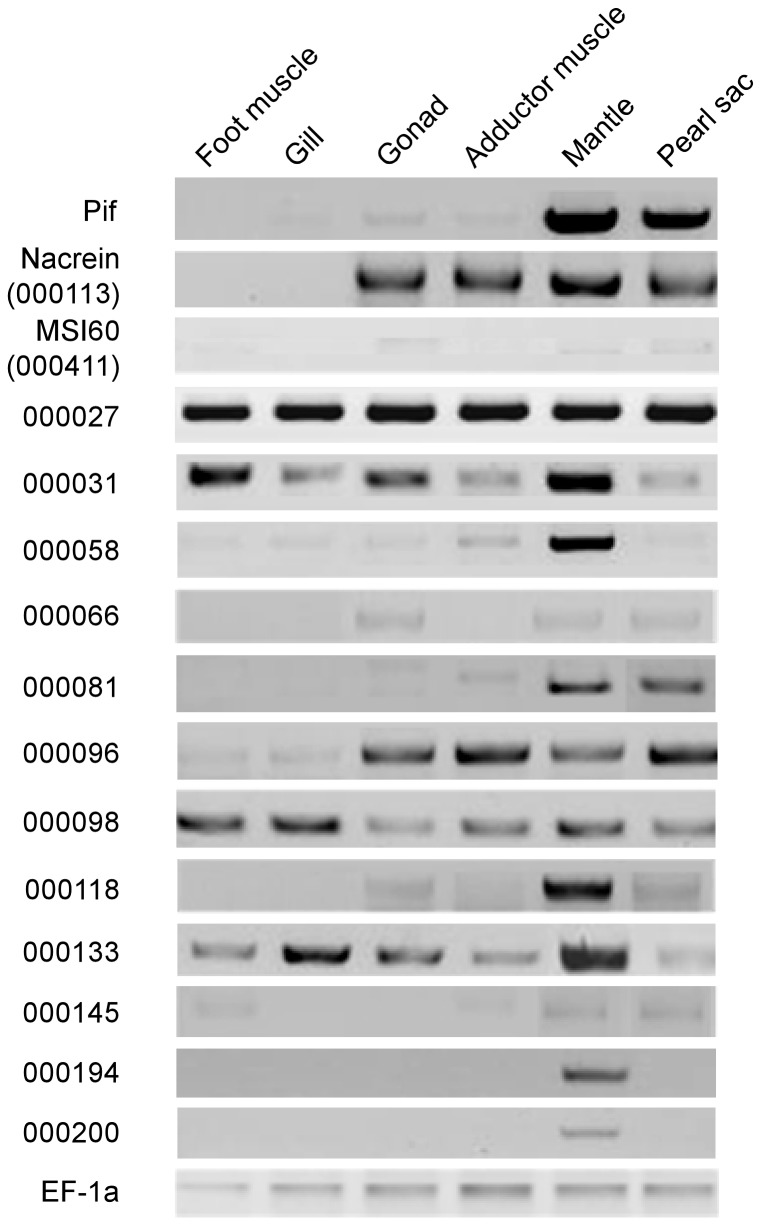
Tissue specific gene expression patterns analyzed by RT-PCR. The elongation factor 1-α gene (*EF-1α*) was used as the internal control.

**Figure 3:**
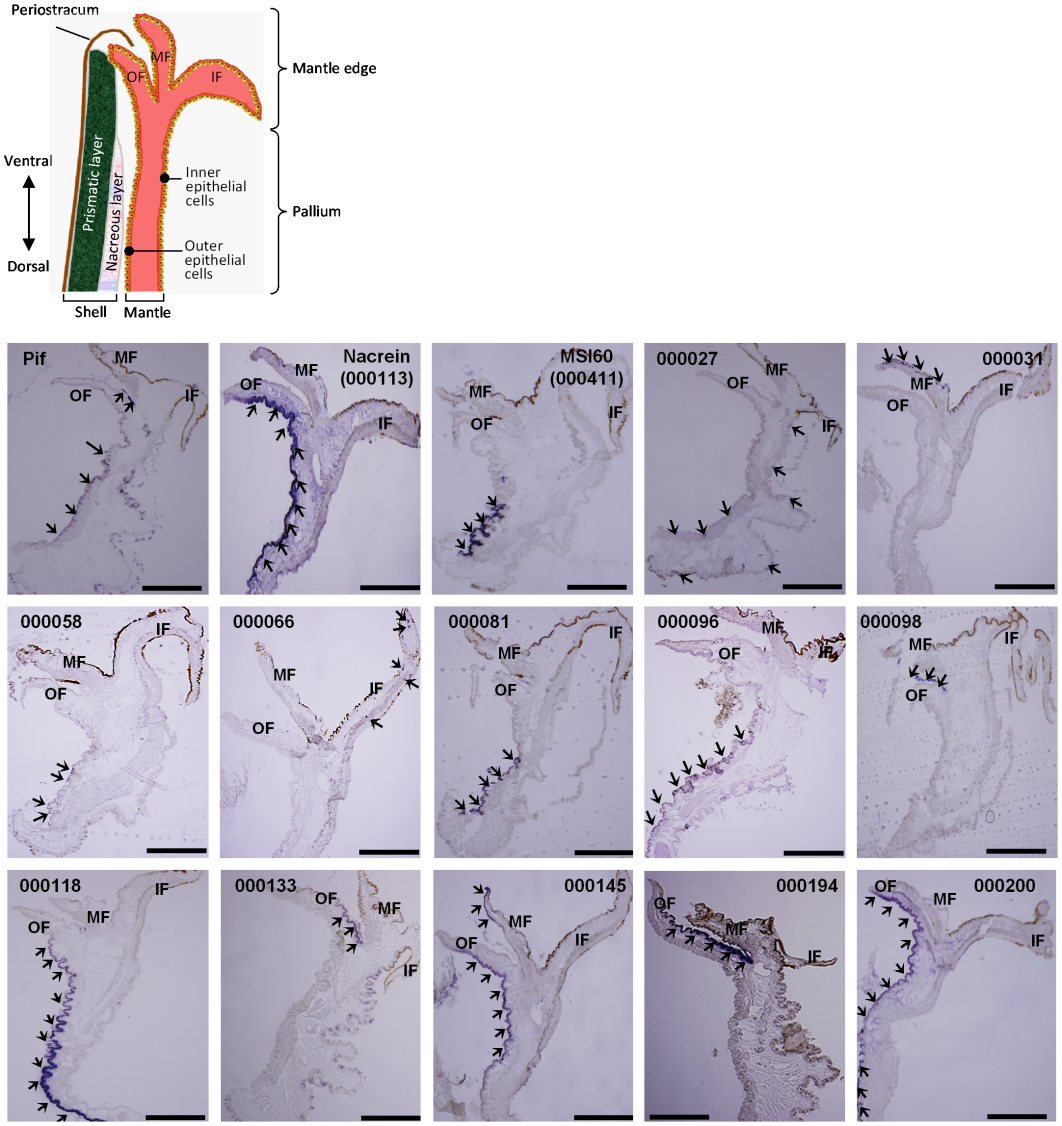
Localization of gene transcripts in the mantle of the pearl oyster. Arrowheads in the photographs indicate transcripts detected by *in situ* hybridization. Scale bars indicate 0.5 mm. Abbreviations used are: OF, outer fold; MF, middle fold; IF, inner fold.

RT-PCR and *in situ* hybridization were performed to examine tissue specificity and regional distribution in the mantle of transcripts encoding the target genes. *Pif* is a well known nacreous gene of *P. fucata* [6] and was used as the positive control. RT-PCR confirmed that *Pif* was expressed in the mantle and pearl sac (Fig. 2), and its transcripts were mainly detected in the outer epithelial cells of the pallium (Fig. 3), supporting the results of our previous report [8].

*Nacrein* (000113) and *MSI60* (000411) are also known nacreous genes. RT-PCR showed that *nacrein* was expressed in the gonad, muscle, mantle and pearl sac, whereas *MSI60* was expressed in the gonad, mantle and pearl sac (Fig. 2). Nacrein mRNA was strongly expressed in the outer epithelial cells of both the pallium and the mantle edge (Fig. 3), whereas MSI60 mRNA was detected only in the outer epithelial cells of the pallium (Fig. 3). These data are consistent with those previously reported [8].

Genes 000081, 000194 and 000200 were detected specifically in shell-forming tissues: the former was detected in both the mantle and pearl sac, whereas the latter two were detected only in the mantle (Fig. 2). During RT-PCR of gene 00081, a weak band was also detected in adductor muscle; however, the size of the detected band was larger than those detected in the mantle and pearl sac. Therefore, the band was considered to be a non-specific amplification product. *In situ* hybridization showed that the transcripts of gene 000081 were specifically distributed to the outer epithelial cells of the pallium, whereas those of gene 000194 were observed in the inner epithelial cells of the outer fold in the mantle edge (Fig. 3). On the other hand, the transcripts of gene 000200 were detected in the outer epithelial cells in both the pallium and the mantle edge (Fig. 3).

Genes 000058 and 000118 were expressed in the mantle and some other tissues (Fig. 2). *In situ* hybridization localized their transcripts in the outer epithelial cells of the pallium (Fig. 3).

Genes 000066, 000096 and 000145 were expressed in shell-forming tissues, as well as non-shell forming tissues, such as the muscle, gonad and gill (Fig. 2). *In situ* hybridization showed that the transcripts of genes 00096 and 000145 were distributed to the outer epithelial cells in the pallium and mantle edge, whereas the transcripts of gene 000066 were localized in the inner fold of the mantle (Fig. 3).

Genes 000027, 000031, 000098 and 000133 were expressed in all tissues analyzed in the present study (Fig. 2), whereas *in situ* hybridization showed a variety of regional expression patterns in the mantle (Fig. 3). The transcripts of gene 000027 were localized in the outer and inner epithelial cells of the pallium, but not of the mantle edge. The transcripts of genes 000031 and 000098 were distributed specifically to the middle fold in the mantle edge. The transcripts of gene 000133 were distributed to the inner epithelial cells of the pallium and mantle edge, and to the outer fold in the mantle edge.

### Effect on Shell Formation of RNAi Knockdown

**Figure 4:**
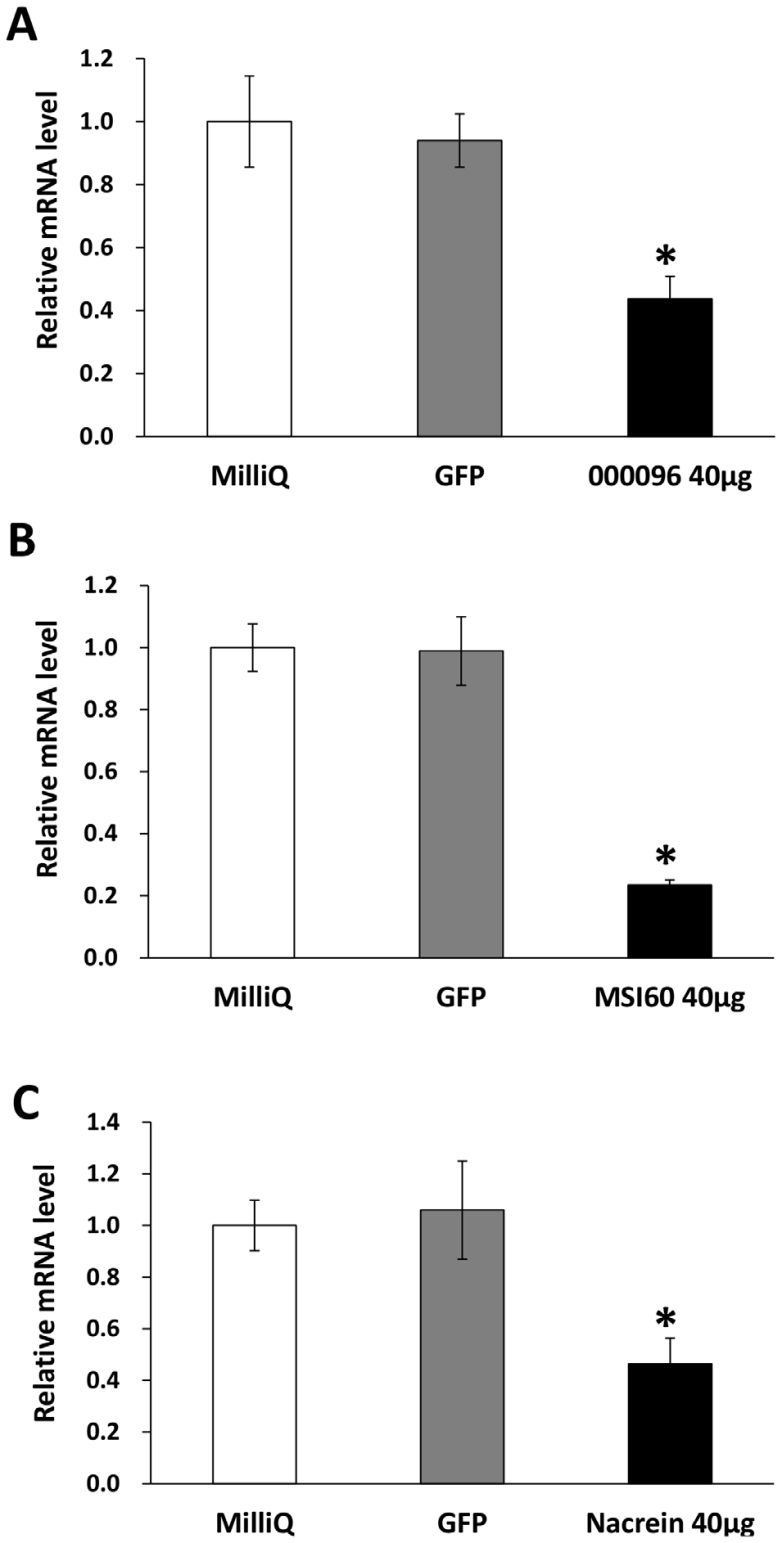
Expression levels of 000096, *MSI60* and *nacrein* in the mantle of the pearl oyster (*Pinctada fucata*) injected with MilliQ water and dsRNAs against GFP and the indicated genes. Expression levels of 000096 (A), *MSI60* (B) and *nacrein* (C) in the mantle were suppressed by RNAi. Each value represents the mean ± SE of three samples each from three individuals. The values are normalized to those of *EF-1α*. For the control, the expression levels of the MilliQ-injected group were set at 1.0. Asterisks represent significant differences (ANOVA, *P*<0.05) compared with the MilliQ-injected group.

We validated the effect of RNAi knockdown on transcriptional expression by qPCR focusing on known genes and one candidate gene, 000096. As shown in Fig. 4, the transcripts of these genes were successfully decreased to 20–40% of the control value after dsRNA injection. Although the expression levels of all the candidate genes after the RNAi experiments were not analyzed, we considered that the RNAi knockdown was successful in all cases because all individuals injected with dsRNA had abnormal appearances, as described below.

**Figure 5:**
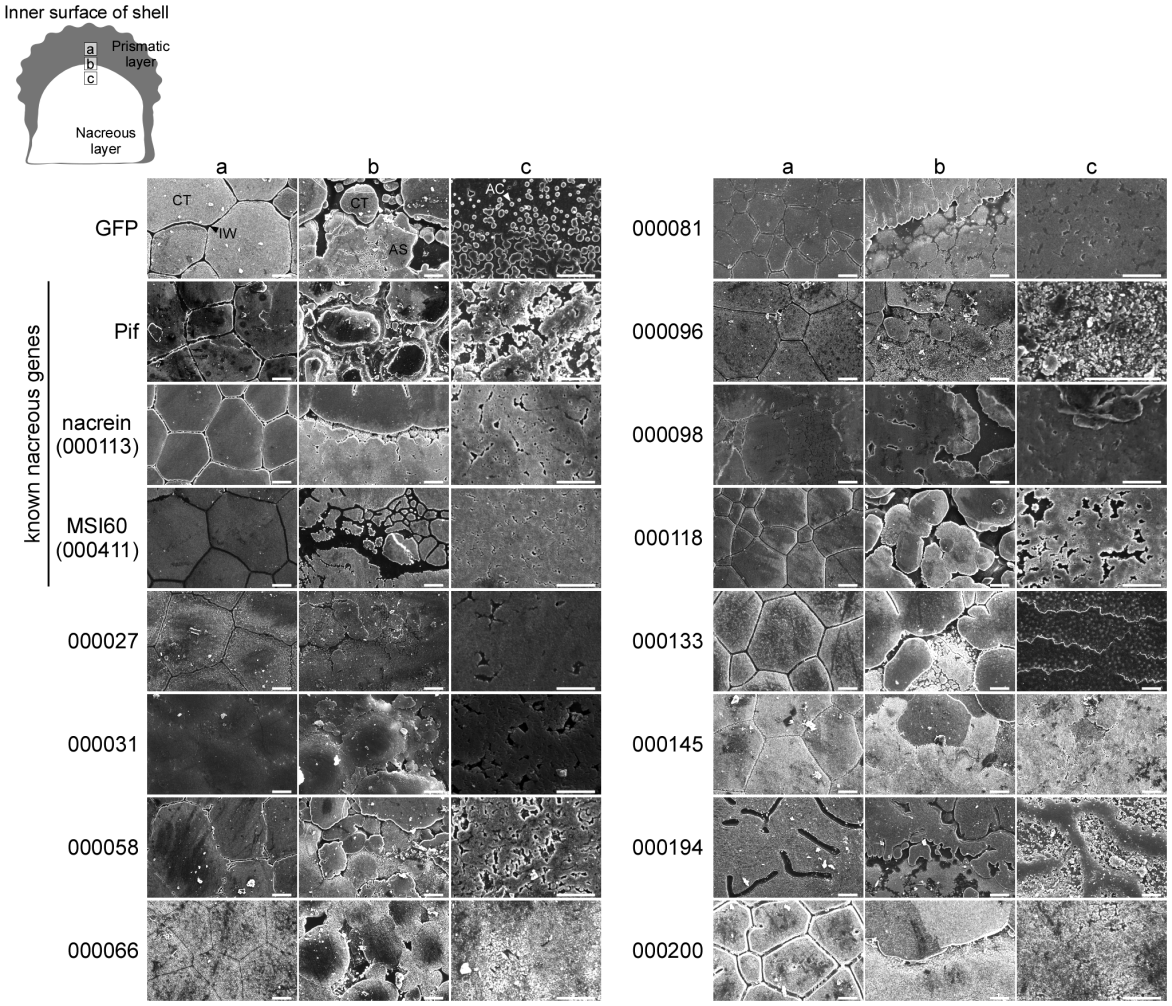
Scanning electron microscopy of the prismatic and nacreous layers, and the boundary between them, in shells of the pearl oyster (*Pinctada fucata*) injected with dsRNAs against the target genes. a, b and c at the top represent the observed positions in the shell as illustrated at the upper left. Scale bars indicate 10 µm. Black and white arrowheads indicate interprismatic wall and aragonite crystals, respectively, in photographs of shells of the GFP dsRNA injected oyster. CT, calcite tablet; IW, interprismatic wall; AS, aragonite sheet; AC, aragonite crystal.

RNAi knockdown phenotypes in the shell were observed at three positions: the prismatic layer (position a in Fig. 5), the boundary of the prismatic and nacreous layers (position b) and the nacreous layer (position c). The typical prismatic layer of the wild-type pearl oyster comprises calcite tablets in the shape of a polygon separated from each other by the interprismatic walls. The nacreous layer comprises many small aragonite crystals in the shape of hexagons that form striated patterns after they aggregate together, a typical characteristic of the nacreous layer. Injection of GFP dsRNA had no effects on the phenotype of either the prismatic or the nacreous layer (Fig. 5).

As well as expressional analyses, we used *Pif* as the control gene to verify our experiments. The RNAi knockdown of *Pif* induced an abnormal appearance of the nacreous layer similar to that reported previously [6]. Although the prismatic layer showed calcite tablets with a normal shape, the interprismatic walls that are supposed to form the calcite tablets were slightly disordered (Fig. 5). The boundary region between the calcite and aragonite crystals contained growing calcite tablets, the appearance of which was different from that observed after injecting GFP dsRNA (Fig. S1).

*Nacrein* and *MSI60* are known nacreous genes; however, their RNAi knockdown phenotypes have not been reported yet. *Nacrein* and *MSI60* knockdown oysters had normal patterns of calcite tablets on the prismatic layer, as in the case of the GFP treated oyster (Fig. 5). However, the interprismatic walls in a *nacrein* knockdown individual looked spongy, as if some components were not present. Abnormal patterns appeared on the nacreous layer after RNAi knockdown of the two genes, where no aragonite crystals with rectangle shapes were observed, but with many grooves and holes. The calcite-aragonite boundary was occupied with abnormal nacreous layers (See also Fig. S1).

As well as *Pif*, *nacrein* and *MSI60*, genes 000027, 000081, 000096 and 000118 were predominantly or specifically expressed in the pallium (Fig. 3, Table S1), suggesting roles in the formation of the nacreous layer. Actually, the effects of RNAi knockdown of genes 000081, 000096 and 000118 on the phenotype were observed only in the nacreous layer, whereas RNAi knockdown of gene 000027 affected both the prismatic and nacreous layers. The details of the phenotypes are as follows.

The 000027 knockdown oyster was not able to form the normal prismatic and nacreous layers (Fig. 5). Newly produced calcite crystals seemed to be unable to grow correctly into tablets, resulting in the failure to construct the interprismatic walls, with various shapes of calcite crystals on the prismatic layer. Furthermore, aragonite crystals disappeared, with many holes on the nacreous layer (See also Fig. S1).

The shape of calcite tablets in the 000081 knockdown oyster appeared to be almost normal, with the interprismatic walls being slightly disordered (Fig. 5). In contrast, the nacreous layer showed an abnormal appearance: aragonite crystals were absent and many small holes were observed (Fig. S1). The translation product of gene 000081 was predicted to have a signal peptide, three transmembrane helices and two glycosylation sites (Fig. 1), suggesting that the 000081 protein is a membrane protein that contributes to transportation of the substances required to form the nacreous layer.

RNAi knockdown of gene 000096 had no effect on the prismatic layer, where typical calcite tablets were well arranged, whereas the appearance of the nacreous layer was completely different from the wild-type (Fig. 5). Numerous particles with very small sizes were observed on the nacreous layer (Fig. S1).

The prismatic layer of the 000118 knockdown oyster showed a normal appearance, with calcite tablets tightly arranged with normal interprismatic walls (Fig. 5). Growing calcite tablets were observed near the calcite-aragonite boundary, whereas the appearance of the aragonite crystals looked abnormal. No typical crystals were observed in the calcite-aragonite boundary area and striation patterns had completely disappeared (Fig. S1).

Gene 000133 was expressed twice as much in the pallium as in the mantle edge (Table S1); however, *in situ* hybridization only detected its expression in the outer fold of the mantle edge (Fig 3). The explanation for this discrepancy between the expression profiles detected by the two methods is unknown. RNAi knockdown of gene 000133 changed the appearance of the nacreous layer completely (Fig. 5). Although the shape of the calcite tablets looked quite normal, the interprismatic walls disappeared near the boundary region between the calcite and aragonite crystals. Very abnormal patterns, like fish scales, appeared in the middle region of the shell. These scales were easily chipped away from the surface (Fig. S1).

Genes 000031, 000066, 000145, 000194 and 000200 were predominantly or specifically expressed in the mantle edge (Table S1). We predicted initially that these genes would be involved only in the formation of the prismatic layer, because of their regional expression in the mantle. However, RNAi knockdown of these genes affected the formation of both the nacreous and prismatic layers. Details of phenotypes produced by RNAi knockdown of these genes are as follows.

In the 000031 and 000066 knockdown oysters, the interprismatic walls disappeared in the prismatic layer and the calcite tablets were fused to each other (Fig. 5). The nacreous layer also looked abnormal. The 000031 knockdown individuals showed no growing aragonite crystals and the 000066 knockdown individuals showed no aragonite crystals in the supposed nacreous layer (Fig. S1).

The 000145 knockdown oysters showed apparently normal prismatic layers (Fig. 5), whereas calcite tablets near the boundary were abnormal in shape, and several tablets were fused to each other, resulting in the disappearance of the interprismatic walls. The nacreous layer was also abnormal and showed no granules on their surface (Fig. S1).

The prismatic layer in the 000194 knockdown oyster showed an abnormal surface (Fig. 5). The shapes of the calcite tablets were disordered and the interprismatic walls had disappeared. The appearance of the nacreous layer was also abnormal, showing a mountain ridge shape with many small particles (Fig. S1).

The prismatic layer of the 000200 knockdown oyster showed a different appearance from typical calcite crystal patterns (Fig. 5). There was no interprismatic wall near the calcite-aragonite boundary. Numerous cracks were observed all over the surface of the shell. It was hard to distinguish the nacreous layer from the prismatic layer because of the presence of abnormal crystals with similar appearances (Fig. S1).

Although genes 000058 and 000098 showed predominant expression in the mantle edge and pallium, respectively, by next generation sequencing (Table S1), *in situ* hybridization showed the opposite regional expression of these genes in the mantle (Fig. 3). RNAi knockdown of these genes caused abnormal phenotypes in both the prismatic and nacreous layers.

The prismatic layer of the 000058 knockdown oyster showed abnormal calcite tablets with their interprismatic walls (Fig. 5). The nacreous layer was also abnormal, showing no aragonite crystals. On the other hand, RNAi knockdown of gene 000098 resulted in the loss of the interprismatic walls (Fig. 5). In comparison with the individual injected with GFP dsRNA, it was difficult to distinguish prismatic and nacreous layers because their boundary region was obscure, with abnormal calcite and aragonite crystals (Fig. S1). The nacreous layer looked flat with many holes.

## Discussion

In the present study, we identified 12 novel genes that are responsible for shell formation, using RNAi methods, RT-PCR and *in situ* hybridization. The fact that the RNAi knockdown of each gene resulted in abnormal shell formation phenotypes confirmed that our previous screening of gene candidates for shell formation based on the expression levels of genes in the EST data [8] was successful.

RT-PCR showed that certain genes were expressed in tissues other than those of the mantle and pearl sac (Fig. 2). The transcripts of genes 000133, 000098, 000027 and 000031 were observed in the foot muscle, gill, gonad, adductor muscle and mantle. The transcripts encoded by genes *Pif*, *nacrein* (000113), 000118 and 000096 were expressed in the gonad and adductor muscle, in addition to the mantle and pearl sac. These observations imply that tissues other than the mantle and the pearl sac may also be necessary for shell formation. Alternatively, these genes may have different functions in tissues where no shells are formed.

It has been reported that shell matrix proteins, such as nacrein from the pearl oyster *P. fucata*, calprismin and mucoperlin from the Mediterranean fan mussel *Pinna nobilis*, dermatopontin from the freshwater snail *Biomphalaria glabrata* and MSP-1 from the scallop *Patinopecten yessoensis*, undergo glycosylation, which is thought to play a key role in shell formation [22]–[27]. The present study revealed five of the predicted secretory proteins (000031, 000081, 000098, 0000194 and 000200) have potential glycosylation sites (Fig. 1), implying that they are contained in the shell matrix as glycoproteins.

Initially, we speculated that genes specifically expressed in the tissues forming the nacreous layer such as the pallium and pearl sac, in our EST data [8] were responsible for nacreous layer formation. This speculation proved correct: RNAi experiments showed that knockdown of these genes specifically expressed in the pallium and pearl sac resulted in an abnormal appearance, mainly in the nacreous layer. We similarly speculated that genes specifically expressed in tissues that form the prismatic layer, such as the mantle edge, would be responsible for the formation of the prismatic layer. Such speculation was also proven to be true by the RNAi experiments. Unexpectedly, these genes also appear to be involved in the formation of the nacreous layer. RNAi knockdown of these genes resulted in severely abnormal appearances in the nacreous layer, raising the possibility that genes responsible for the formation of the prismatic layer might also be involved in the formation of the nacreous layer. Marie et al. (2012) demonstrated that different repertoires of proteins are involved in the formation of the nacreous and prismatic layers, respectively, suggesting that the two layers are not derived from each other [28]. They showed that the composition of shell matrix proteins in the prismatic layer, which may be secreted from the mantle edge, is clearly different from that in the nacreous layer, which may be secreted from the pallium. The abnormality of the appearances of the prismatic and nacreous layers might result from a changed ratio of gene expression caused by gene knockdown.

Genes specific to mantle edge, each of which was subjected to RNAi knockdown in the present study, were not expressed in the pearl sac (Table S1). If the hypothesis described above is true, the pearl sac must have no ability to form the nacreous layer, because genes 000031, 000058, 000066, 000145, 000194 and 000200, considered to be responsible for the nacreous layer formation, are not expressed there. However, in our previous report, we confirmed that the pearl oyster subjected to EST analysis, data from which were used in the present study, produced a beautiful pearl in a pearl sac, with a normal nacreous layer surrounding the nucleus [8]. Why does the pearl sac produce the nacreous layer, even though genes involved in the nacreous layer formation are absent? One possibility is that the shell and pearl sac form the nacreous layer using different molecular mechanisms. The nacreous layer comprises calcium carbonate in the form of aragonite crystals and an organic matrix in which several components have been identified. Our previous investigation showed that gene expression patterns were different between the pallium and the pearl sac [8], [18], [29], implying that their protein compositions are not identical. This hypothesis has been partly demonstrated by SDS-PAGE of matrix proteins extracted from the nacreous layer of the shell and pearl (unpublished data).

Some RNAi knockdown individuals provided a common phenotype of the nacreous layer, as shown in SEM images of Fig. 5. The RNAi knockdown of nacrein (000113), MSI60 (000411), gene 000027, gene 000031 and gene 000081, which all have signal peptides except for 000027, resulted in nacreous layers with similar appearances. This raised the possibility that they participate in the formation of the nacreous layer in cooperation in the same process. As shown in Fig. 1 and Table 1, BLAST searching showed that gene 000027 is a putative ribosomal protein, which may be involved in normal cell metabolism. RT-PCR showed that it is expressed in all tissues (Fig. 2) and broadly in mantle by in situ hybridization (Fig. 3), suggesting that the product of gene 000027 may be a housekeeping protein. Although knockdown of gene 000027 caused the abnormal appearance of the nacreous layer, gene 000027 may not be involved directly in shell-formation. The RNAi knockdown of Pif, gene 000058 and 000118 also resulted in the abnormal nacreous layer with a common phenotype. These genes are possibly involved in the formation of the layer in a different process from that mentioned above. No other common phenotype was found in the nacreous layer. On the other hand, there were no common phenotypes among the prismatic layers of the RNAi knockdown individuals.

**Figure 6:**
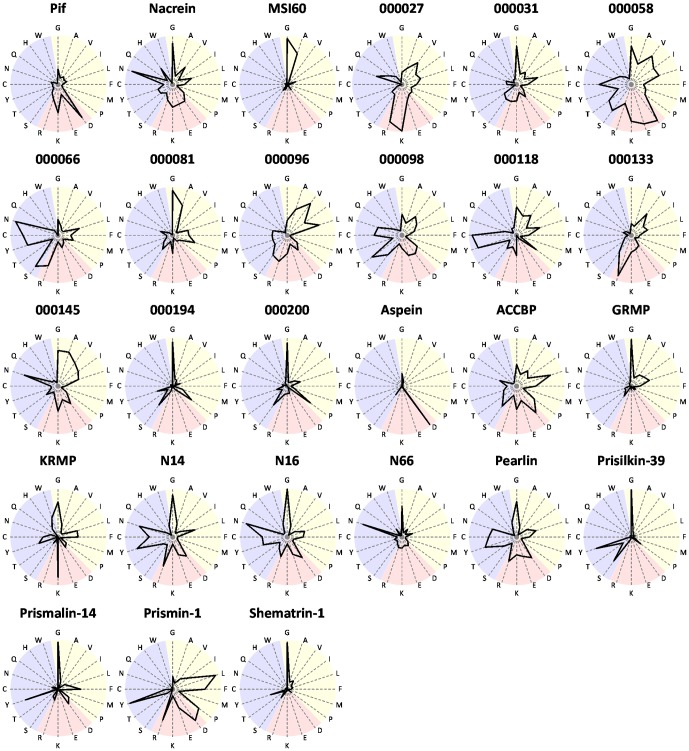
Amino acid composition of deduced amino acid sequences from candidate genes for shell formation in this study and proteins registered in the database as shell formation-related proteins. The letters on around the graphs represent amino acids. Yellow, red and blue regions represent hydrophobic, charged and polar amino acids, respectively. Data of known genes were cited from the GenBank: Pif (BAH97338), nacrein (BAA11940), MSI60 (BAA20466), aspein (AB094512), ACCBP (DQ473430), GRMP (AF516712), KRMP (DQ114788), N14 (Q9NL39), N16 (BAL46657), N66 (AB032613), pearlin (BAA75626), prisilkin-39 (EU921665), prismalin-14 (AB159512), prismin-1 (AB368930) and shematrin-1 (AB244419).

For further prediction of the function of candidate genes in this study, we compared the amino acid composition of the gene products with those of shell-formation related proteins reported to date (Fig. 6).

The protein encoded by 000081 has a glycine-rich sequence and showed clear similarity with MSI60 in the shape of star diagram. Genes 000081 and *MSI60* also showed similar expression patterns, where transcripts of the two genes were found in the pallium and pearl sac (Fig. 3). In addition, the RNAi knockdown phenotypes of the two genes showed similar abnormality in the nacreous layer (Fig. 5), suggesting a functional relationship in the shell formation.

Proteins encoded by genes 000194 and 000200 had similarly shaped star diagram, where glycine is dominant. Their shapes resembled those of prisilkin-39 [30] and shematrin-1 [31]. Prisilkin-39 is a matrix protein in the prismatic layer, has been reported to be expressed in the mantle edge and is considered to be involved in the prismatic layer formation [30]. The shematrin gene was cloned from a cDNA library constructed from mRNAs of the mantle by random sequencing [31]. Shematrin is predicted to be secreted from the mantle edge into the prismatic layer to participate in calcification. As stated in the results section, BLAST searching of genes 000194 and 000200 identified no homologous genes in the database including those encoding prisilkin-39 and shermatri-1 (Table 1). However, the similarity in the amino acid compositions of these proteins may reflect their similar functions in shell formation to some extent. The disordered interprismatic walls in the prismatic layer resulting from RNAi knockdown of genes 000194 and 000200 (Fig. 5) imply that their encoded proteins play important roles in the formation of the framework for calcite tablets, which is coincident with hypothetical function of prisilkin-39 and shematrin-1. Although there is no homology in protein structure (Figs. 1 and 6), it is also notable that similar phenotypes were observed in the nacreous layer by RNAi knockdown of genes 000200 and *nacrein* (Fig. 5). *In situ* hybridization also showed the same distribution of transcripts of the two genes in the mantle (Fig. 3), suggesting their cooperative function in nacreous layer formation.

It is reasonable to find similarity in the star diagram between nacrein and N66, which was isolated from shell matrix of *P. maxima*, because they are homologous proteins [2], [32]. The Gly-Xaa-Asn repeat domain of nacrein and N66 is thought to contribute to their interaction with calcium to form shells. As described above, we showed that the RNAi knockdown of nacrein produced abnormal nacreous layers (Fig. 5). These results provide useful information to understand the function of N66 in shell formation.

The proteins encoded by genes 000066 and 000118 are rich in tyrosine and cysteine (Fig. 6), which are considered to be involved in polymerization of proteins secreted from mantle, resulting in the formation of matrix sheets in shells [33]. The two proteins are predicted to have signal peptides (Fig. 1), indicating that they are accumulated as shell matrix proteins after secretion from the mantle. Pearlin and N16 belong to the same protein family and are known as shell matrix proteins rich in tyrosine and cysteine [34], [35]. The proteins encoded by genes 000066 and 000118 might participate in the formation of protein networks with other secreted proteins, yielding insoluble protein sheets. RNAi knockdown of genes 000066 and 000118 caused disappearance of the interprismatic walls and aragonite crystals, and abnormal aragonite crystal structure, respectively (Fig. 5). These phenotypes suggest that they encode components of aragonite crystals and/or interprismatic walls. The two proteins are basic, with p*I* values of 8.90 and 8.67, respectively (Table 1), whereas those of pearlin and N16 are 6.10 and 4.68, respectively, before glycosylation. The different p*I*s are caused by low aspartic and glutamic acids contents in the two molecules (Fig. 6).

The proteins encoded by genes 000058 and 000098 are relatively large among proteins encoded by the present novel genes and both are acidic (Table 1) and rich in cysteine (Fig. 6). No protein with such properties has been found in those used for comparison. The RNAi knockdown of gene 000058 showed indistinct structure of the interprismatic walls in the prismatic layer and that of gene 000098 showed reduced walls (Fig. 5). These data indicate that proteins encoded by the two genes might be involved in constructing the framework of calcite tablets via disulfide bonds formed with other proteins.

In the present study, we identified novel genes involved in shell formation, based on the alteration of the surface appearance of the nacreous and prismatic layers induced by RNAi. It should be noted that it is possible that all of these knockdowns have had absolutely no specific effect on protein levels because this has not been measured with antibodies. It is therefore possible that all of the phenotypes we observe are non-specific or general off-target effects. To eliminate those possibilities, further study is needed on the proteins encoded by the genes in shell formation; for example, tissue distribution analysis of proteins with antibodies, and *in vitro* precipitation assays of aragonite crystals in the presence of artificially expressed proteins encoded by the genes could be conducted.

## Supporting Information

Figure S1 **Scanning electron microscopy of the boundary between the prismatic and nacreous layers in the shells of the pearl oyster** ***Pinctada fucata*** **injected with dsRNAs of target genes.** The prismatic layer starts from the top of the photo and end in the middle of the photo. A red arrowhead in each photo shows the boundary between the prismatic and nacreous layers identified by the shape of calcite tablets. Scale bars indicate 30 µm.
(TIF)

Table S1 **Genes selected in the present study.**
(PDF)

Table S2 **Sequences of gene specific primers used in 5′-RACE.**
(PDF)

Table S3 **Sequences of gene specific primers used in RT-PCR.**
(PDF)

Table S4 **Sequences of gene specific primers used in in situ hybridization.**
(PDF)
