# Supplementary figures and images for: Novel Genes Participating in the Formation of Prismatic and Nacreous Layers in the Pearl Oyster as Revealed by Their Tissue Distribution and RNA Interference Knockdown
Source: PLoS One. 2014 Jan 15;9(1):e84706. doi: 10.1371/journal.pone.0084706 (PMC3893171; doi:10.1371/journal.pone.0084706)

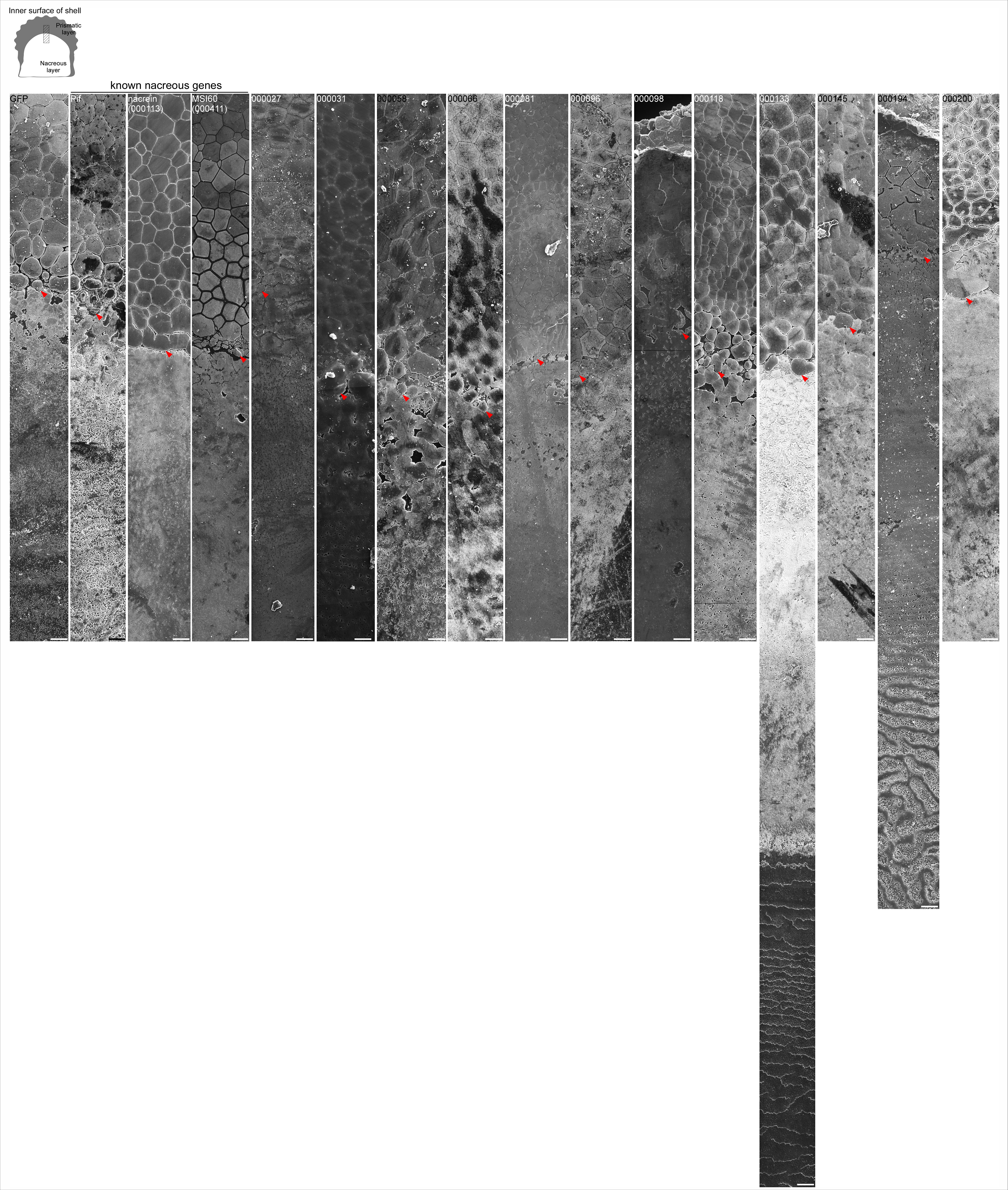

Supplement: Figure S1 — Scanning electron microscopy of the boundary between the prismatic and nacreous layers in the shells of the pearl oyster Pinctada fucata injected with dsRNAs of target genes. The prismatic layer starts from the top of the photo and end in the middle of the photo. A red arrowhead in each photo shows the boundary between the prismatic and nacreous layers identified by the shape of calcite tablets. Scale bars indicate 30 µm. (TIF) [file pone.0084706.s001.tif]
