# Supplementary material for: Novel Genes Participating in the Formation of Prismatic and Nacreous Layers in the Pearl Oyster as Revealed by Their Tissue Distribution and RNA Interference Knockdown
Source: PLoS One. 2014 Jan 15;9(1):e84706. doi: 10.1371/journal.pone.0084706 (PMC3893171; doi:10.1371/journal.pone.0084706)
Supplement: Table S1 — Genes selected in the present study. (PDF) [file pone.0084706.s002.pdf]

Table S1. Genes selected in the present study

	Gene	Appearance frequency (10^{-3}) ME	P	PS	Total reads
P specific	000081	0.36	1.63	0.46	211
	000098	1.19	2.76	0.19	334
	000113 (nacrein)	1.02	2.65	0.06	298
	000118	0.64	1.92	0.10	216
	000133	0.90	1.80	0.05	218
P & PS specific	000027	0.64	1.74	3.43	561
	000096	0.44	1.45	2.70	443
	000411 (MSI60)	0.13	2.11	0.26	214
ME specific	000031	4.59	1.27	0.00	421
	000058	2.64	0.27	0.00	204
	000066	2.85	0.05	0.00	200
	000072	3.03	0.14	0.00	292
	000104	1.92	0.91	0.15	224
	000145	4.05	0.17	0.00	292
	000194	2.75	1.03	0.00	275
	000200	2.23	0.79	0.00	219

Abbreviations used are: ME, mantle edge; P, pallium; PS, pearl sac.
