# Supplementary material for: Novel Genes Participating in the Formation of Prismatic and Nacreous Layers in the Pearl Oyster as Revealed by Their Tissue Distribution and RNA Interference Knockdown
Source: PLoS One. 2014 Jan 15;9(1):e84706. doi: 10.1371/journal.pone.0084706 (PMC3893171; doi:10.1371/journal.pone.0084706)
Supplement: Table S2 — Sequences of gene specific primers used in 5′-RACE. (PDF) [file pone.0084706.s003.pdf]

Table S2. Sequences of gene specific primers used in 5’-RACE

Target Gene	Primer name	Primer sequence
000027	000027-R1	5'-TGGCTTACGCCAGTTAGCCT-3'
000031	000031-R1	5'-TGTCCAGAGACGTGAACAGT-3'
	000031-R2	5'-GGGTCATCATGTCTGAACCA-3'
000058	000058-R1	5'-TGTTTCGGGAGAGTCAGAAT-3'
	000058-R2	5'-GGTACTATTTCACCGGTCAT-3'
000066	000066-R1	5'-CGTGGGACCAGTAGTAGTTA-3'
000081	000081-R1	5'-CCACCACCTCCAGTCATACT-3'
000098	000098-R1	5'-GGTTTACACACAGGACAACA-3'
	000098-R2	5'-GGGAGAGGATCGGTACAACT-3'
000118	000118-R1	5'-GACTGCAAGCGATGTACAAG-3'
000133	000133-R1	5'-CTCGAGGTAACCGAACAATT-3'
000194	000194-R1	5'-CGGCGATGGACTGCCATAGT-3'
	000194-R2	5'-CGGGTGCTCTGTAGTAGGAT-3'
000200	000200-R1	5'-GGTGTCCGCCATGATGATAA-3'
	000200-R2	5'-TATCCAGAGCCAGAATACCG-3'
