# Supplementary material for: Novel Genes Participating in the Formation of Prismatic and Nacreous Layers in the Pearl Oyster as Revealed by Their Tissue Distribution and RNA Interference Knockdown
Source: PLoS One. 2014 Jan 15;9(1):e84706. doi: 10.1371/journal.pone.0084706 (PMC3893171; doi:10.1371/journal.pone.0084706)
Supplement: Table S3 — Sequences of gene specific primers used in RT-PCR. (PDF) [file pone.0084706.s004.pdf]

Table S3. Sequences of gene specific primers used in RT-PCR

Target gene	Primer sequence		Amplicon size (bp)
	Forward	Reverse	
000027	5'-TCCCCGATTGAGGTCCGCAT-3'	5'-CTTCAGACCTAAGACGAGCA-3'	438
000031	5'-TGGTCAACAGGAGTCGGAG-3'	5'-TCTCCTCGCCGACCACCTTT-3'	517
000058	5'-TGACCCGAAGGCGTCATGCA-3'	5'-TGTTTCGGGAGAGTCAGAAT-3'	581
000066	5'-CCCTTACCGTATCCAAGAAA-3'	5'-TGGGACATCAACCACAGTCT-3'	562
000081	5'-CGGCGCACCGTCACGATATT-3'	5'-GCACAGCACACAGAGAATCT-3'	789
000096	5'-GCGGCCGCACTAGTGATTAA-3'	5'-CTAGAGCACTCGTATGCAAT-3'	411
000098	5'-TGTTGTCCTGTGTGTAAACC-3'	5'-TTCTTCTTCCTGGTGCTGTA-3'	303
Nacrein (000113)	5'-GACTAGGTGCGCTAACCCCT-3'	5'-GGCCCCATTTTCAAAGTTGT-3'	511
000118	5'-CCGTGTCCAAACTCATTATCT-3'	5'-GCATACGCCATACACACATT-3'	344
000133	5'-GAATTGTTCGGTTACCTCGA-3'	5'-GCCCAACCAGAGACAGCCTT-3'	516
000145	5'-GGGAAGAGAGAATGGCAGGA-3'	5'-GGCTACAGGATGCAAATGAA-3'	347
000194	5'-CATGGACTGGAAGGAGTAGA-3'	5'-TGGGGCCTTGTTCGTCTTTT-3'	1331
000200	5'-TATCCAGAGCCAGAATACCG-3'	5'-GCCATACTCAATACGACAAA-3'	976
MSI60 (000411)	5'-CAGGTGGTGGAGGAAGAGCA-3'	5'-GCCGTTATTTCCCCATCCAT-3'	559
Pif	5'-ACACCGACTCATGGTTCCGA-3'	5'-ATCGCCCGTGTGTTGCTTGA-3'	1258
EF-1α	5'-GGCCACAGAGATTTCATCAAGAAC-3'	5'-CAACACCAGCAGCAATAATCAACAC-3'	82
