# Supplementary material for: Novel Genes Participating in the Formation of Prismatic and Nacreous Layers in the Pearl Oyster as Revealed by Their Tissue Distribution and RNA Interference Knockdown
Source: PLoS One. 2014 Jan 15;9(1):e84706. doi: 10.1371/journal.pone.0084706 (PMC3893171; doi:10.1371/journal.pone.0084706)
Supplement: Table S4 — Sequences of gene specific primers used in in situ hybridization. (PDF) [file pone.0084706.s005.pdf]

Table S4. Sequences of gene specific primers used in *in situ* hybridization

Target gene	Primer name	Primer sequence	Amplicon size (bp)
000027	000027-F	5'-TAATACGACTCACTATAGGGTACTCCCCGATTGAGGTCCGCATTG-3'	395
	000027-R	5'-ATTTAGGTGACACTATAGAATCTTCAGACCTAAGACGAGCATTAG-3'	
000031	000031-F	5'-TAATACGACTCACTATAGGGTTCGTGGTCAACAGGAGTCGGAGAC-3'	515
	000031-R	5'-ATTTAGGTGACACTATAGAATTTGTCTCCTCGCCGACCACCTTTG-3'	
000058	000058-F	5'-TAATACGACTCACTATAGGGGTGACCCGAAGGCGTCATGCAACAG-3'	645
	000058-R	5'-ATTTAGGTGACACTATAGAAAATAATCAGGACTACTGGCCATTTC-3'	
000066	000066-F	5'-TAATACGACTCACTATAGGGGCGCCCAGTTCTCTAACTACTACTG-3'	352
	000066-R	5'-ATTTAGGTGACACTATAGAAATGCAGCACCTTGGATTACATATAG-3'	
000081	000081-F	5'-TAATACGACTCACTATAGGGAGGTCAAGATGAATCTGATGAAAAG-3'	495
	000081-R	5'-ATTTAGGTGACACTATAGAAGCACAGCACACAGAGAATCTATTAAAC-3'	
000096	000096-F	5'-TAATACGACTCACTATAGGGGCAGAGCTGCCCCTGTCATCTCAAC-3'	297
	000096-R	5'-ATTTAGGTGACACTATAGAAAGATCCTTCACTACACCATAGGCAG-3'	
000098	000098-F	5'-TAATACGACTCACTATAGGGAACGGCGTAAACTATACATCCGGAG-3'	435
	000098-R	5'-ATTTAGGTGACACTATAGAAGAATCTGCACATAAATTGCAACCAC-3'	
000118	000118-F	5'-TAATACGACTCACTATAGGGACGCGACCAATTCACGTTAAATACC-3'	430
	000118-R	5'-ATTTAGGTGACACTATAGAAAGTAAGCATATCCCCTTTTGCATAC-3'	
000133	000133-F	5'-TAATACGACTCACTATAGGGGGAGTCCGTAAAGTTTTCCGAAGTG-3'	670
	000133-R	5'-ATTTAGGTGACACTATAGAAGAGTTTCGTGTCCTCAGTACATTGTC-3'	
000145	000145-F	5'-TAATACGACTCACTATAGGGAGGTATCACTATTTTGTCTGGCTGC-3'	648
	000145-R	5'-ATTTAGGTGACACTATAGAACTTAGCGAAAAGTGAAGAGAACTAG-3'	
000194	000194-F	5'-TAATACGACTCACTATAGGGATTCTCCAGGTTCTGGCCCTGCAGG-3'	634
	000194-R	5'-ATTTAGGTGACACTATAGAATAGCCATACCGCTACCCATACCATC-3'	
000200	000200-F	5'-TAATACGACTCACTATAGGGGCTTATCCCACCGAGTGAAAAGCAC-3'	410
	000200-R	5'-ATTTAGGTGACACTATAGAACCACTGCCCATTCCTCCGCCCATTC-3'	
Nacrein	Nacrein-F	5'-TAATACGACTCACTATAGGGACTAGGTGCGCTAACCCCTTATATC-3'	506
	Nacrein-R	5'-ATTTAGGTGACACTATAGAACATATCGGCCCCATTTTCAAAGTTG-3'	
MSI60	MSI60-F	5'-TAATACGACTCACTATAGGGCAGGTGGTGGAGGAAGAGCACTTAG-3'	552
	MSI60-R	5'-ATTTAGGTGACACTATAGAAGTTGCCGTTATTTCCCCATCCATTG-3'	
Pif	Pif-F	5'-TAATACGACTCACTATAGGGGACACCGACTCATGGTTCCGAACTG-3'	515
	Pif-R	5'-ATTTAGGTGACACTATAGAATGCTCTCTATCTCGTCTCTGTCTAC-3'	

Underlines indicate T7 or SP6 RNA polymerase binding site.
